# New-phase retention in colloidal core/shell nanocrystals *via* pressure-modulated phase engineering†

**DOI:** 10.1039/d1sc00498k

**Published:** 2021-04-02

**Authors:** Yixuan Wang, Hao Liu, Min Wu, Kai Wang, Yongming Sui, Zhaodong Liu, Siyu Lu, Zhihong Nie, John S. Tse, Xinyi Yang, Bo Zou

**Affiliations:** State Key Laboratory of Superhard Materials, College of Physics, Jilin University Changchun 130012 China yangxinyi@jlu.edu.cn zoubo@jlu.edu.cn; Green Catalysis Center, College of Chemistry, Zhengzhou University Zhengzhou 450001 China sylu2013@zzu.edu.cn; State Key Laboratory of Molecular Engineering of Polymers, Department of Macromolecular Science, Fudan University Shanghai 200438 China; Department of Physics and Engineering Physics, University of Saskatchewan Saskatoon Saskatchewan S7N 5E2 Canada

## Abstract

Core/shell nanocrystals (NCs) integrate collaborative functionalization that would trigger advanced properties, such as high energy conversion efficiency, nonblinking emission, and spin–orbit coupling. Such prospects are highly correlated with the crystal structure of individual constituents. However, it is challenging to achieve novel phases in core/shell NCs, generally non-existing in bulk counterparts. Here, we present a fast and clean high-pressure approach to fabricate heterostructured core/shell MnSe/MnS NCs with a new phase that does not occur in their bulk counterparts. We determine the new phase as an orthorhombic MnP structure (B31 phase), with close-packed zigzagged arrangements within unit cells. Encapsulation of the solid MnSe nanorod with an MnS shell allows us to identify two separate phase transitions with recognizable diffraction patterns under high pressure, where the heterointerface effect regulates the wurtzite → rocksalt → B31 phase transitions of the core. First-principles calculations indicate that the B31 phase is thermodynamically stable under high pressure and can survive under ambient conditions owing to the synergistic effect of subtle enthalpy differences and large surface energy in nanomaterials. The ability to retain the new phase may open up the opportunity for future manipulation of electronic and magnetic properties in heterostructured nanostructures.

## Abstract 2

Core/shell MnSe/MnS nanocrystals with the B31 phase are thermodynamically stable under high pressure and can survive under ambient conditions owing to the synergistic effect of subtle enthalpy differences and high surface energy in nanomaterials.

## Graphical abstract

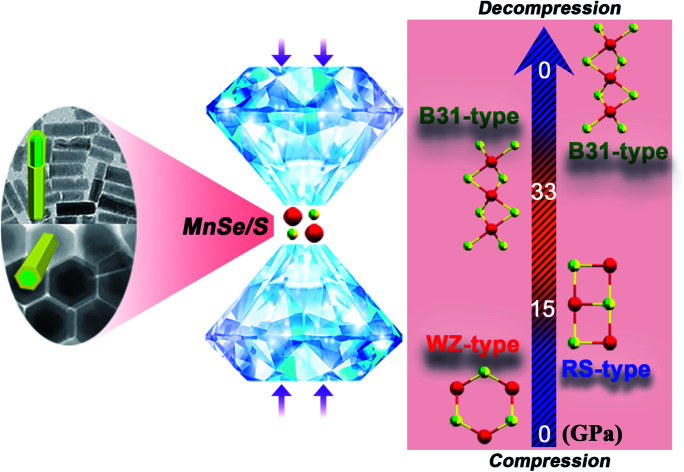

## Introduction

Heterostructured core/shell and heterojunction nanocrystals (NCs) have emerged as an interesting class of materials because of their unique optical, electronic, catalytic and magnetic properties originating from their individual constituents.^1–6^ The combination and synergistic effect of multiple components within one particle often result in new or advanced properties of NCs, such as high energy conversion efficiency, high photoluminescence quantum yield, nonblinking emission, and spin–orbit coupling.^7–11^ Core/shell NCs are usually constructed through the epitaxial growth of a second material onto the surface of a seed nanoparticle in a set of configurations (*e.g.*, dot/dot, dot/rod, rod/rod, and wire/wire forms).^12–15^ Among others, one-dimensional (1D) core/shell NCs are attractive and suitable for various target applications that are otherwise difficult to achieve with individual NCs or isotropic heterostructures.^16^ In this regard, the form of crystal phases is equally if not more important than the morphology of NCs. To date, an array of classic semiconductors (*e.g.*, CdS, CdSe, and ZnS) and III–V materials have been used to fabricate 1D coaxial core–shell heterostructures.^17–19^ However, these core–shell heterostructures are usually composed of well-known conventional phases that are existed in the corresponding bulk counterparts.^20^ The search for 1D core/shell NCs with unusual crystal phases is essential for the development of novel phase-dependent properties and materials, yet their controllable synthesis and delicate modulation remain elusive.

Pressure-induced phase engineering offers opportunities for the rational design and synthesis of materials with unusual crystal phases, in particular, unconventional high-pressure new phases.^21,22^*In situ* pressure-processing has been considered as a fast and clean mechanical method for the fabrication of nanomaterials with a controlled morphology and phase without involvement of chemical reactions and post purification processes.^23–25^ Unlike solution-phase synthesis, the high-pressure technique allows for monitoring structure modulation of nanomaterials under continuous compression, offering direct evidence for the structural stability and transition process of materials.^26^ Inspired by the pressure-induced structural phase transition of CdSe nanoparticles, significant advances in high-pressure nanotechnology have been accomplished by tuning the morphology, construction and crystal structure.^27–30^ However, relatively few studies have been reported for high-pressure nanophases that could be anticipated to survive under ambient conditions, in particular unconventional high-pressure new phases.

In this work, we undertake a study on the high-pressure phase transition behaviors of heterostructured core/shell MnSe/MnS nanorods, especially focusing on the new-phase retention engineering of core/shell nanostructures. The results of this study indicate that high pressure could trigger wurtzite (WZ) → rocksalt (RS) → B31 phase transitions in the core/shell MnSe/MnS nanorods by a combination of high-pressure angle dispersive X-ray diffraction (ADXRD) and high-resolution transmission electron microscopy (HRTEM) characterization studies as well as first-principles calculations. Furthermore, the generated new phase core/shell nanorods were captured as expected by quenching the high-pressure phase under ambient conditions at room temperature.

## Results and discussion

### Fresh heterostructured core/shell MnSe/MnS nanorods

**Fig. 1:**
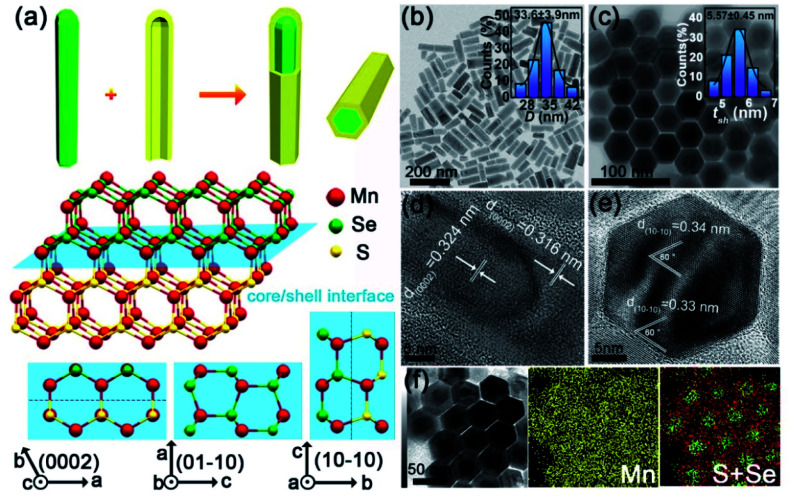
Synthesis and characterization of heterostructured core/shell MnSe/MnS nanorods with the WZ structure. (a) Top: schematic illustration of the synthesis of heterostructured core/shell MnSe/MnS nanorods. After the WZ MnSe nanorod is synthesized, it is used as a seed for the crystal-phase-based epitaxial growth of the WZ MnS nanoshell. The growth direction of the MnS nanoshell on the MnSe nanorod is [0001]_WZ_. Middle: the crystal structure of WZ-type core/shell MnSe/MnS. Bottom: the views of the core/shell interface in different directions. (b) TEM image of synthesized core/shell MnSe/MnS nanorods; inset: histograms showing the distribution of core/shell nanorod diameter, *D*. (c) TEM images of the self-assembled nanorods; inset: histograms showing the distribution of the shell thickness, *t*_sh_. (d and e) HRTEM images of synthesized core/shell MnSe/MnS nanorods. (f) STEM elemental map of heterostructured core/shell MnSe/MnS nanorods. The green dot represents Se and the red dot represents S.

We synthesized heterostructured MnSe/MnS core/shell nanorods with the WZ structure using a solvothermal method. The synthesis involves two main steps (Fig. 1a): (I) the preparation of high-quality WZ MnSe nanorods (∼24 nm × 75 nm) (Fig. S1†) and (II) the coaxial growth of the WZ MnS shell on MnSe nanorods. The core–shell structure and morphological uniformity of nanorods were confirmed by transmission electron microscopy (TEM) imaging (Fig. 1b and c), *i.e.* the aspect ratio is ∼2.5, with a length of 85.1 ± 11.7 nm and a width of 33.6 ± 3.9 nm (the inset of Fig. 1b and S2†), where the MnS shell was measured to be *t*_sh_ = 5.57 ± 0.45 nm (the inset of Fig. 1c). As shown in the top-view TEM images, the morphology of rods did not undergo noticeable changes before and after the growth of MnS layers. High-resolution TEM (HRTEM) images reveal the different lattice plane motifs, providing details about the representative atomistic-structure information and microtopography of the coaxial core–shell nanorods. The periodicity of the fringes of the core is 0.324 nm, corresponding to the (002) plane of hexagonal MnSe (Fig. 1d).^31^ The MnS shell shows a fringe spacing of 0.316 nm that matches the (002) plane of hexagonal MnS.^32^ The top-view HRTEM images indicated the smooth growth of MnS on MnSe and the explicit coaxial core–shell structure rather than the sulfur doped MnSe pattern (Fig. 1e). Energy-dispersive X-ray spectroscopy (EDS) mapping further confirmed the core–shell nanostructure with the Se element in the center and S element in the periphery (Fig. 1f).

### Pressure-induced phase transition and formation of the new phase

**Fig. 2:**
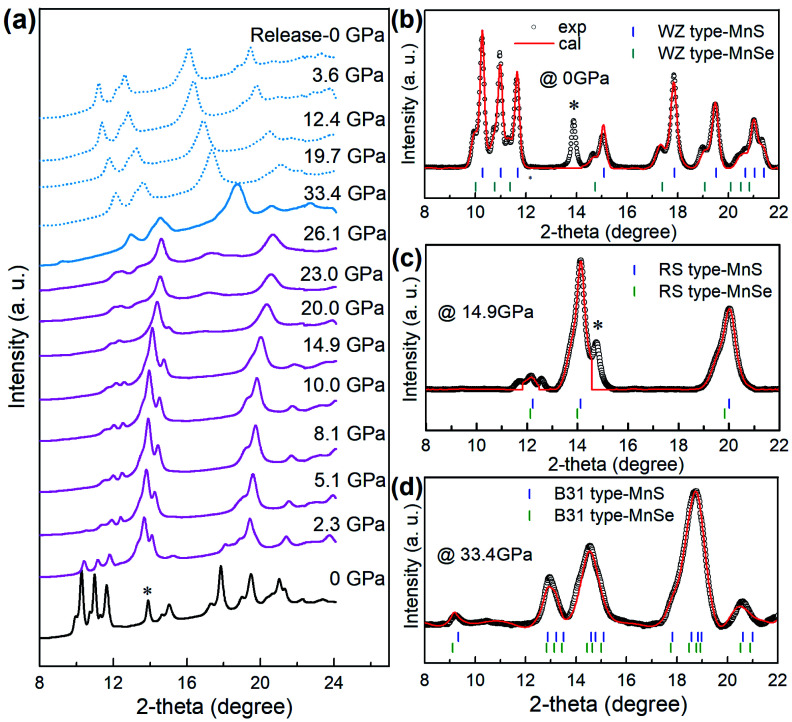
Pressure-induced structural evolution of heterostructured core/shell MnSe/MnS nanorods during compression and decompression processes. (a) Representative *in situ* ADXRD patterns of core/shell MnSe/MnS nanorods during the high-pressure experiments. (b–d) Rietveld refinements of the experimental (black fork) and simulated (red profile) ADXRD patterns of the WZ phase at 0 GPa, RS phase at the pressure of 14.9 GPa, and the B31 phase at the pressure of 33.4 GPa. Blue and green vertical markers indicate the corresponding Bragg reflections. Black hexagram markers in (a–c) show the diffraction peak of SeO_2_.

We performed high-pressure treatment of core/shell MnSe/MnS nanorods at a pressure up to 33.4 GPa and monitored the phase transition and formation of the new phase by *in situ* synchrotron angle dispersive X-ray diffraction (ADXRD) measurements (Fig. 2a). Before compression, the ADXRD pattern indicated that the core–shell nanostructures showed two sets of diffraction patterns that correspond to the planes of WZ MnS and MnSe, respectively (Fig. 2b). The superimposing of diffraction peaks provides further evidence that the NCs are composed of core/shell structures. When WZ core/shell nanorods were compressed under pressure above 2.3 GPa, the intensity of original diffraction peaks decreased drastically and signals of the RS phase gradually enhanced (Fig. 2c). The two sets of diffraction peaks shifted to higher angles, indicating the pressure-induced lattice shrinkage of both WZ and RS structures. Prominent structural transitions from the RS phase to a new high-pressure phase occurred at approximately 20.0 GPa, as indicated by the appearance of a distinctive new peak at 16.9° that cannot be assigned to any previously known phases in bulk MnS or MnSe materials. The new phase remained stable to about 33.4 GPa. The new phase at 33.4 GPa is indexed and can be refined to the B31 structure (orthorhombic, *Pnma*) of MnS and MnSe, respectively (Fig. 2d) using Rietveld refinement. We investigated the WZ-to-RS-to-B31 transition mechanism by monitoring the pressure dependence of lattice parameters. We found that the lattice constants decreased with the increase of pressure and exhibited abrupt changes (Fig. S3†) at 2.3 and 26.1 GPa, suggesting two first-order phase transitions. Notably, the lattice volume reduces significantly by about 35% when core/shell nanostructures transited from WZ to B31 phases. For most materials, a 5% volume collapse during a phase transition can be regarded as notable. It is suggested that the giant pressure-driven lattice collapses can be attributed to the formation of Mn–Mn intermetallic bonds and the Mn^2+^ spin state from high-spin (*S* = 5/2) to low-spin (*S* = 1/2).^33^ Such lattice collapse driven by adjustable orbital/spin-state responses is rare. This unusual phenomenon should be further explored to accelerate the structural design of novel functional materials.^33^

### Structure of B31-type core/shell MnSe/MnS nanorods

**Fig. 3:**
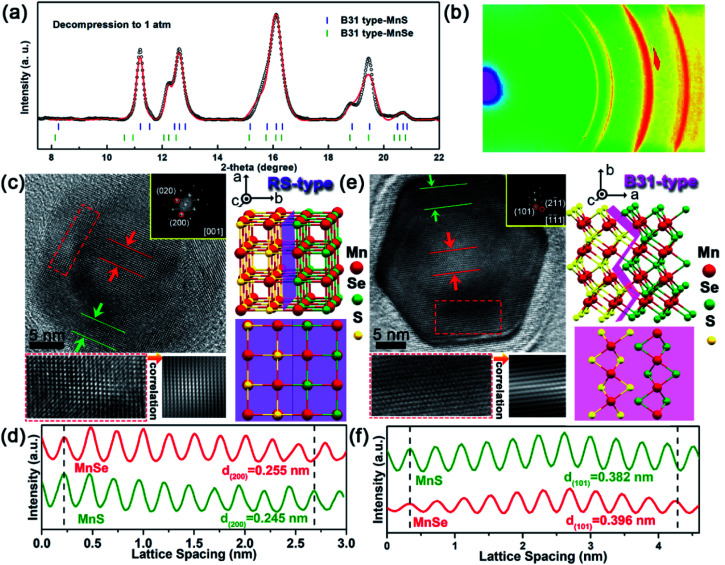
Synthesis and characterization of heterostructured core/shell MnSe/MnS nanorods with high-pressure phases. (a) Rietveld refinements of the experimental (black circle) and simulated (red profile) ADXRD patterns of core/shell samples with the B31 phase decompressed from 33.4 GPa to 1 atm. Blue and green vertical markers indicate the corresponding Bragg reflections. (b) The corresponding 2D ring-type ADXRD pattern. (c) Left: HRTEM images of RS-type core/shell MnSe/MnS nanorods decompressed from 18.0 GPa to 1 atm. Correlation patterns taken from the corresponding dashed red rectangle. Right: the crystal structure of RS-type core/shell MnSe/MnS, viewed along the [001]_R_ zone axis. Partial purple highlights display the RS-type core/shell interface feature. (d) The integrated pixel intensities along the arrow directions of the corresponding selected areas in the middle (red line) and side (green line) of the RS-MnSe/MnS nanorods shown in (c). The peaks and valleys stand for the alternating atoms and spaces, respectively. (e) Left: HRTEM images of B31-type core/shell MnSe/MnS nanorods decompressed from 33.4 GPa to 1 atm. Correlation patterns taken from the corresponding dashed red rectangle. Right: the crystal structure of B31-type core/shell MnSe/MnS, viewed along the [−1−11]_B_ zone axis. Partial pink highlights display the B31-type core/shell interface feature. (f) The integrated pixel intensities along the arrow directions of the corresponding selected areas in the middle (red line) and side (green line) of the B31-MnSe/MnS nanorods shown in (e).

Upon releasing pressure, the positions of diffraction peaks shift back to lower angles due to the decompression-induced volumetric expansion of the crystal structures (Fig. 2a). Note that the new high-pressure phase can be retained after completely releasing the pressure to ambient conditions. The well-fitted refinements of the quenched ADXRD pattern indeed confirmed that the recovered new phase is an orthorhombic B31-type polymorph with a space group of *Pnma* (Fig. 3a). The experimental lattice parameters of MnSe with the B31 phase under ambient conditions are *a* = 5.902 (1) Å, *b* = 3.900 (3) Å, and *c* = 6.500 (5) Å. Moreover, lattice parameters of B31-type MnS were estimated to be *a* = 5.656 (1) Å, *b* = 3.662 (3) Å, and *c* = 6.344 (5) Å, which are highly consistent with the previous results.^34^ The two sets of ring-type diffraction patterns in the ADXRD pattern correspond to the planes of B31-type MnS and MnSe, indicating the entrapment of the core–shell nanostructures (Fig. 3b).

TEM images provided additional evidence for the formation of coaxial core/shell MnSe/MnS nanorods with the high-pressure phase and preservation of their subsequent dual structure (Fig. S4†). In principle, two new types of 1D core/shell MnSe/MnS NCs were obtained: (1) RS-type nanorods and (2) B31-type nanorods. Representative HRTEM images in Fig. 3c show characteristic array lattice fringes of the single domain shell and the encapsulated core of the heterorods depressurized from 18.0 GPa compression. The continuous lattice fringes across the interface between the MnSe core and MnS shell indicate the integrality of the core/shell nanorod configuration. Selected-area fast Fourier transform (FFT) patterns match well with the typical [001]_R_-zone axis diffraction pattern of the RS phase, exhibiting the diffraction spots of (020)_R_ and (200)_R_ planes (inset of Fig. 3c). Fig. 3d shows the integrated pixel intensities of MnSe (200)_R_ and MnS (200)_R_ lattices from the selected areas indicted in Fig. 3c. The average interlayer spacing of the MnSe (200)_R_ planes is calculated to be 2.55 Å (red lines), which is slightly (*ca.* 4.1%) greater than that of the MnS (200)_R_ planes (2.45 Å, green lines). Since the B31-type structure obtained during the RS-to-B31 phase transformation at high pressure is energetically more stable, the irreversible transformation in decompression is supported by the HRTEM and the corresponding FFT patterns. The atomic structure of the B31-type MnSe core is surrounded by the B31-type MnS shell suggesting that both the new high-pressure phase and core/shell geometry were captured after the diamond anvil cell was recovered to the ambient conditions at room temperature (Fig. 3e). The selected-area FFT pattern from the top-view HRTEM image is consistent with the characteristic [−1−11]_B_ zone axis diffraction pattern of the B31 phase, demonstrating the diffraction spots of the (101)_B_ and (2−11)_B_ planes (inset of Fig. 3e). As indicated by the dashed red rectangle in Fig. 3e, there are smooth interfaces between two different materials, and a well-defined core/shell structure of the B31 phase along the close packed directions of [−1−11]_B_ is formed. Fig. 3f shows the integrated pixel intensities of MnS (101)_B_ and MnSe (101)_B_ lattices from the selected areas in Fig. 3e. The average interlayer spacing of the MnS (101)_B_ planes was calculated to be 3.82 Å (green lines), which is slightly (*ca.* 3.5%) less than that of the MnSe (101)_B_ planes (3.96 Å, red lines). Based on the aforementioned results, a structural model of the as-prepared core/shell MnS/MnSe nanorods with the B31 phase is schematically illustrated on the right side of Fig. 3e. The retention of the core–shell nanostructure of decompression samples was further confirmed by the dark-field scanning transmission electron microscopy (STEM) image and corresponding energy-dispersive X-ray spectroscopy (EDS) elemental mapping (Fig. S5†). This manifests that new structured NCs could be accessed through an irreversible structural phase transition in response to high pressure compression.

### Optical properties and correlation with the structure

**Fig. 4:**
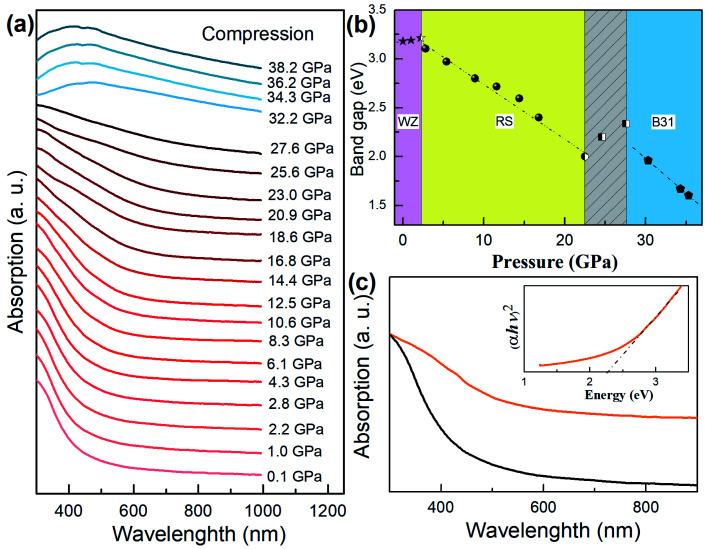
Optical evolution of heterostructured core/shell MnSe/MnS nanorods during pressure-induced phase transition processes. (a) *In situ* high-pressure UV-vis-NIR absorption spectra of heterostructured core/shell MnSe/MnS nanorods. (b) Typical profile of band gaps against pressures for core/shell MnSe/MnS nanorods measured *in situ* in a DAC apparatus. Therein, dashed lines represent the corresponding linear fitting toward different regions. (c) UV-vis-NIR absorption spectrum of the synthesized WZ-type core/shell MnSe/MnS nanorods (black line) and B31-type core/shell MnSe/MnS nanorods (orange line). Inset depicts the plot of (*αhv*)^2^*versus hv* according to Kubelka–Munk transformations.

The optical properties of core/shell NCs can be correlated with the phase transition during compression and pressure release (Fig. 4a). We translated the band gap *E*_g_ in terms of Kubelka–Munk transformations:(*αhν*)^2^ = *A*(*hν* − *E*_g_)where *A* and *hν* are the edge–width parameter and the incident photon energy, respectively.^35^ We found that the band gaps of core/shell NCs decrease over the compression cycle (Fig. 4b). When pressure is increased above ∼2.8 GPa, the band gap narrows abruptly. We speculate that this is caused by the observed phase transition from the direct band gap WZ to the indirect band gap RS. However, when the pressure reached roughly 22.6 GPa, the band gap of the compressed samples suddenly increased. This stark change indicates the onset of the phase transition evolving from RS to B31 structures. The RS phase present in mixed phases gradually vanished when the pressure approached a transition pressure of about 28.3 GPa, and the band gap decreased upon further compression to 34.8 GPa. We proposed that the phase transformation to the B31 structure was completely achieved above 28.3 GPa. Upon complete release of the pressure, a new absorption edge appears with a band gap of 2.30 eV (Fig. 4c). As discussed above, this new structure can be attributed to the B31-type core/shell MnSe/MnS nanorods, exhibiting a distinct energy narrowing by 0.73 eV in comparison with the WZ-type counterpart (3.03 eV) (Fig. S6†). The temperature dependence of the magnetization measured in an applied field of 500 Oe clearly sheds light on the magnetic properties of B31-type MnSe/MnS nanorods (Fig. S7†). In comparison to the WZ-type MnSe/MnS nanorods with a paramagnetic characteristic from 0–300 K, the B31-type MnSe/MnS nanorods show antiferromagnetic behavior with a Néel temperature of 132 K. Such a Néel temperature is relatively high in Mn-based semiconductor nanomaterials,^36,37^ which are expected to have potential applications in information storage, the emerging field of spintronics, and sensors.

### First-principles calculations

**Fig. 5:**
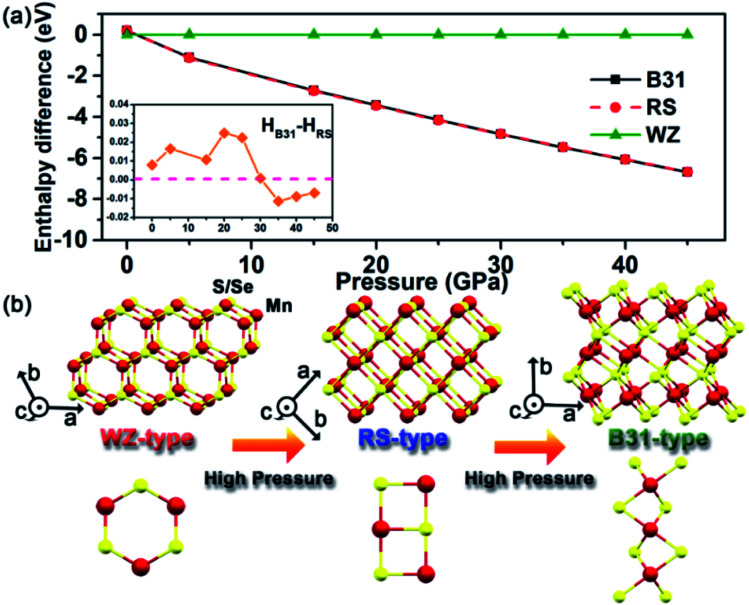
Enthalpy calculations and pressure-induced atomic motions. (a) Theory calculations on energy differences among the WZ, RS and B31-phase MnSe covering the range up to 50.0 GPa. (b) Unit-cell schematics of MnS(Se) with *P*6_3_*mc*, *Fm*3̄*m* and *Pnma* crystal structures under high pressure.

Pressure-driven phase transitions offer us a pivotal protocol to fabricate new-phase core/shell nanoarchitectures. To better understand and interpret our experimental findings, we performed *ab initio* simulation package (VASP) and density functional theory (DFT) calculations on the energy differences among WZ, RS and B31-phase MnSe within the pressure range up to 50.0 GPa (Fig. 5a). The results show that the energies of the three phases are very close under ambient conditions. A pressure-induced phase transition from WZ to RS is predicted at low pressure (<1 GPa). At high pressure, the RS and B31 phases become more stable and the enthalpy difference is comparable to that of the WZ phase and increases with pressure. The enthalpies of RS and B31 are very close to each other at all pressures. A closer examination as shown in the inset of Fig. 5a revealed that phase B31 is more stable above 30.0 GPa. In the MnS system, our previous calculations indicated that the B31 phase is the most stable structure relative to the RS phase under high pressure, whereas the energy of the B31-type structure was very close to that of the RS phase below 8.0 GPa.^34^ Pressure plays an important role in shifting the stability of different structures and thus is a unique tool to create a high-coordination environment for novel materials. The dynamic stability of the new B31 structure was examined by calculating the phonon spectra using the supercell method (Fig. S8†). No imaginary phonon frequencies were found in the entire Brillouin zone over the studied pressure range. This indicates the dynamic stability that favours the reservation of the metastable phase at ambient pressure. We conducted pressure-induced atomic motions to understand the phase transition mechanism. Fig. 5b depicts the schematic illustrations of crystal structures of WZ-, RS- and B31-type MnS(Se) under high pressure. In principle, pressure deforms the four-coordinate WZ into the six-coordinate RS structure without any bond breaking and with very simple atomic displacements, where the main mechanism of the transformation involves the sliding of (100) planes.^38,39^ The transformation pathway from RS to B31 can be considered as the reconstruction between the polyhedral structures, where one Mn atom and six neighboring S(Se) atoms integrally formed a MnS(Se)_6_ octahedron. The MnS(Se)_6_ octahedra are connected by four edges and two vertices for the RS structure, and the adjacent MnS_6_ octahedra in the B31 phase mutually shared the same surface. Thus, the MnS_6_ octahedron in the B31 phase is arranged much more compact than that in the RS phase, which was greatly improved by the creation of zigzag MnS_4_ planes inside the MnS_6_ octahedron along the *b*-axis direction. The rearrangements in the structural buckling profoundly facilitate the phase transformation to accommodate the increased external pressure. Upon decompression, the unique nanostructures and the inevitable thermodynamic influence should be responsible for the retention of the high-pressure new phase by aggravating the fluctuation of the subtle enthalpy differences under atmospheric pressure.

Before clarifying the effect of the MnS shell, we first characterize the crystal structure of pure MnSe NCs under high pressure. In bulk MnSe, the transition sequence during compression has been reported to be RS-to-high-pressure intermediate (HPI) phase-to-B31 up to about 47.4 GPa, where the HPI phase was assigned to a tetragonal distortion structure at an applied pressure of 22.8 GPa.^33^ However, the transitions are unclear during decompression so far. Fig. S9† shows the ADXRD data collected from the sample during compression and pressure release. The results reveal that the phase transformation from initial WZ-MnSe nanorods to the RS phase occurred at about 1.9 GPa. The MnSe NCs then changed into the B31 phase at about 28.0 GPa, where the formation of a HPI phase was assigned to a tetragonal distortion structure at an applied pressure of 18.4 GPa, referring to the structural analysis by Wang *et al.*^33^ After fully releasing the pressure to ambient conditions, pure B31-type MnSe can be obtained.

We further investigated whether the thickness of the MnS shell would affect the aforementioned experimental results. Typically, MnS/MnSe NCs with thin shells (∼3.5 nm) were held between the opposed diamond anvils at room temperature (Fig. S10†). Besides critical pressure points, the phase transition sequence was in accordance with that of the aforementioned core/shell nanostructures with thick shells (Fig. S11†). Compared with pure MnSe nanorods, the WZ-to-RS-to-B31 phase transitions are completely realized at the considerably higher critical pressure for core/shell nanostructures. This behaviour resembles the shell thickness-dependent phase transition pressure associated with the protection of the shell observed in the CdSe/ZnS core/shell system, where it required higher energy for the accomplishment of phase transitions.^40^ On the other hand, the transitions were nucleated on the nanocrystal surface, where sliding and/or flattening of crystal planes occur and proceed inwards with increasing pressure.^36^ For heterostructures, the interface between different structures can act as an initiation site of the core to facilitate the occurrence of pressure-driven solid–solid phase transitions.^23,41^ The large lattice mismatch of the RS-MnS shell and the HPI-MnSe core shows that the absence of HPI in the MnSe core can reduce the interface strain. Therefore, the absence of HPI in the MnSe core may arise from the presence of abundant MnS phase interfaces as the dominant initiation sites, and the comprehensive effects enforced the WZ-to-RS-to-B31 phase transitions.

## Conclusions

In summary, we elucidate a new-phase heterostructured core/shell NC paradigm that is fed back from the pressure-induced B31 phase retention of core/shell MnSe/MnS nanostructures to ambient conditions at room temperature. Structural insights for this phenomenon were obtained using *in situ* ADXRD that identified the WZ-to-RS-to-B31 phase transition path for the core/shell MnSe/MnS nanorods. The generated new high-pressure phase B31-type core/shell NCs were captured as expected by quenching the high-pressure phase to ambient conditions at room temperature. The morphology of core/shell nanorods could be maintained after the high-pressure treatment. First-principles calculations indicate that B31-MnS and MnSe are thermodynamically stable under high pressure, and can survive under ambient conditions owing to the synergistic effect of subtle enthalpy differences in RS and B31 phases and high surface energy in nanomaterials. This study not only provides a fundamental understanding of pressure-driven phase transformations at the atomic scale but also sheds light on the rational design of new-phase heterostructured core/shell nanomaterials through a clean and fast stress-driven nanofabrication technique.

## Author contributions

X. Y. and B. Z. designed the project and supervised the work. Y. W., H. L., M. W., K. W., Y. S., Z. L., Z. N., X. Y. and B. Z. performed experiments and analyzed data. S. L. and J. S. T. performed the calculations.

## Conflicts of interest

There are no conflicts to declare.

## Supplementary Material

SC-012-D1SC00498K-s001
