# Supplementary material for: New-phase retention in colloidal core/shell nanocrystals via pressure-modulated phase engineering
Source: Chem Sci. 2021 Apr 2;12(19):6580–7. doi: 10.1039/d1sc00498k (PMC8133026; doi:10.1039/d1sc00498k)
Supplement: SC-012-D1SC00498K-s001 [file SC-012-D1SC00498K-s001.pdf]

Electronic Supplementary Material (ESI) for Chemical Science.
This journal is © The Royal Society of Chemistry 2021

Supporting Information

New-phase retention in colloidal core/shell nanocrystals via pressure-modulated phase engineering

Yixuan Wang [a] , Hao Liu [a], Min Wu [a], Kai Wang [a], Yongming Sui [a], Zhaodong Liu [a], Siyu Lu *[b], Zhihong Nie [c], John S. Tse [d], Xinyi Yang *[a] and Bo Zou *[a]

[a] State Key Laboratory of Superhard Materials, College of Physics, Jilin University, Changchun, 130012, China

[b] Green Catalysis Center, College of Chemistry, Zhengzhou University, Zhengzhou 450001, China

[c] State Key Laboratory of Molecular Engineering of Polymers, Department of Macromolecular Science, Fudan University, Shanghai 200438, China

[d] Department of Physics and Engineering Physics, University of Saskatchewan, Saskatoon, Saskatchewan S7N 5E2, Canada

* Correspondence author: yangxinyi@jlu.edu.cn; sylu2013@zzu.edu.cn; zoubo@jlu.edu.cn

Supplementary Methods

Chemicals

All chemicals, including SeO_2 powder (99 %) and anhydrous $MnCl_2$ (99 %), thioacetamide (TAA, ≥ 90 %), oleylamine (OLA, ≥ 70 %) and S powder (99 %) were purchased from Aldrich. Toluene and acetone were obtained from commercial sources. All chemicals were received without further purification.

Synthesis of WZ-type core/shell MnSe/MnS nanorods

Firstly, synthesis of WZ-type MnSe nanorods has been reported in our previous studies.[1] Then, 0.050 g (0.4 mmol) $MnCl_2$ and 6 mL of oleylamine was loaded to a 50-mL three-necked flask and heated to 300 ℃ under N_2 flow, then, 2 mL unwashed MnSe product was added. When the solution recovered to 300 ℃, 0.015 g (0.2 mmol) thioacetaminde dissolved in 2 mL oleyamine was added. The reaction was quenched at 5 and 20 mins to get the sample with varied thickness of MnS shell. The resultant WZ-type core/shell MnSe/MnS nanorods were isolated from the growth solution by precipitation with methanol and excess acetone, followed by centrifugation for 10 min at 10000 rpm. Subsequently, the residual samples were redispersed in toluene for characterization.

In situ **high-pressure experimental measurements**

All *in situ* high-pressure experiments were implemented using symmetric DAC apparatus furnished with a pair of 400 μm culet diamonds at room temperature. The prepared core/shell MnSe/MnS nanorods were enclosed into a ~100 μm-diameter hole of the T301 stainless-steel compressible gasket. Silicon oil was utilized as the pressure transmitting medium (PTM) which was purchased from the Dow Corning Corporation (Midland, MI). Pressure determination was achieved by the fluorescence spectrum of the ruby. *In situ* angle dispersive synchrotron X-ray diffraction (ADXRD) patterns of samples under high pressure were recorded at beamline 15U1, Shanghai Synchrotron Radiation Facility (SSRF), China. The beamline stations at SSRF exploited a monochromatic wavelength of 0.6199 Å. CeO_2 was utilized as the standard sample for the calibration. The pattern of intensity versus diffraction angle 2θ was plotted based on the FIT2D program, which integrated and analyzed the 2D images collected. The *in situ* high-pressure UV−vis−NIR absorption spectra were measured by a deuterium-halogen light source and recorded with an optical fiber spectrometer (Ocean Optics, QE65000) at room temperature. Magnetic property measurements were conducted on a Quantum Design MPMS superconducting quantum interference device (SQUID) VSM magnetometer. To guarantee the quantity of samples for magnetic performance, high-pressure experiments on core/shell MnSe/MnS nanorods were carried out in a Walker-type JLUHC-1000 LVP, subsequently by collecting the quenched products for further magnetic characterization.[2]

Theoretical calculations

All the total energy and electronic structure calculations were performed by the Vienna Ab-initio Simulation Package (VASP) that uses the projector augmented wave (PAW) formalism of density functional theory (DFT). The Perdew-Burke-Ernzerhof (PBE) generalized gradient approximation for the exchange-correlation function was employed. The energy cutoff for the plane-wave expansion of the wavefunctions was 400 eV for all the calculations. For highly-correlated systems with 3d localized orbitals, the hybrid functional methods are more appropriate than the general DFT. In the present study, HSE method was employed for both geometry optimization and self-consistent calculation. For geometry optimization, the atomic coordinates were relaxed until the Hellmann-Feynman forces were less 0.01 eV/ Å. In order to compare the stability of MnSe under pressure, three structural models of MnSe with phases of WZ, B31 and RS were used. The pressure dependent enthalpies show that the phase WZ is more stable than phase B31 and RS at low pressure (< 1.0 GPa). At high pressure, phase B31 and RS become more stable and the enthalpy difference compared to phase WZ increases with pressure. The enthalpies of phase B31 and RS are very close to each other at all the calculated pressure. However, by taking a closer examination, it can be found the phase B31 is less stable at pressure lower than 30.0 GPa and becomes more stable above 30.0 GPa.

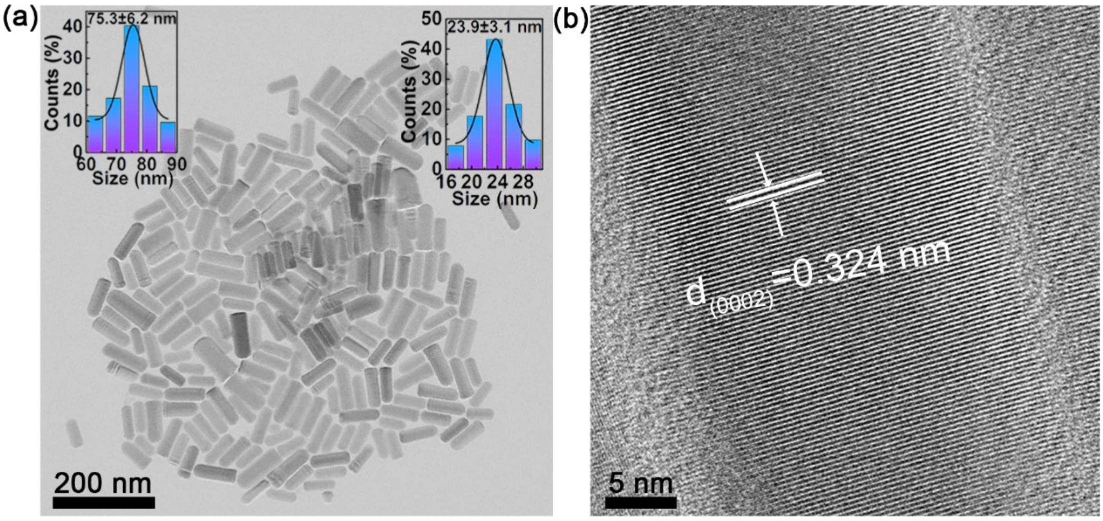

Fig. S1 TEM and HRTEM images of WZ-type MnSe nanorods. (a) The TEM image depicts the high uniform size of the as-prepared MnSe nanorods with a length of 75.3 ± 6.2 nm and a width of 23.9 ± 3.1 nm, based on the analysis of 50 nanorods. (b) High-resolution TEM image indicates that the synthesized MnSe nanorods possess good crystallinity. The interplanar distance of 0.324 nm is shown by HRTEM, which can index as the (0002) crystallographic facet of WZ-type MnSe nanorods.

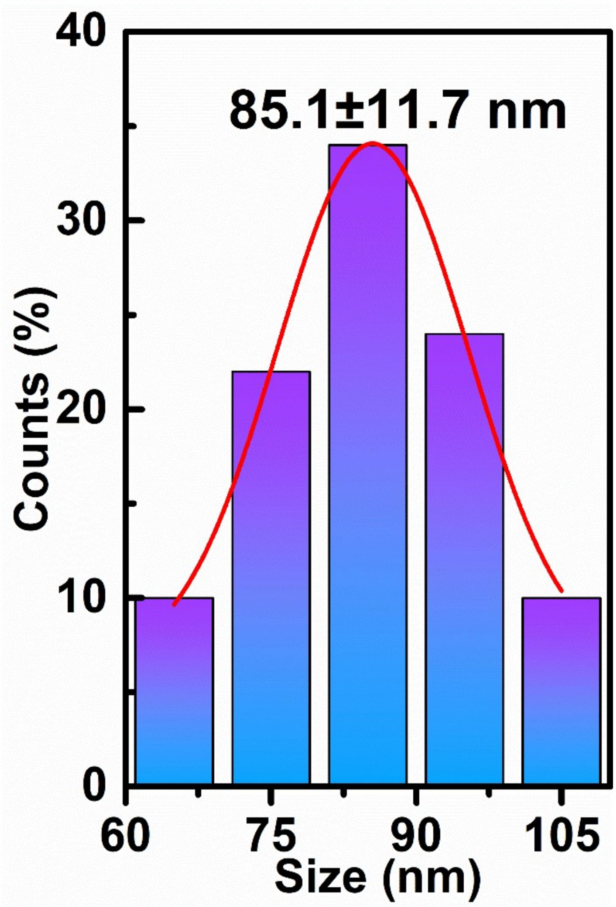

Fig. S2 The length distribution of WZ-type core/shell MnSe/MnS nanorods. The as-synthesized WZ-type core/shell MnSe/MnS nanorods have lengths of 85.1 ± 11.7 nm.

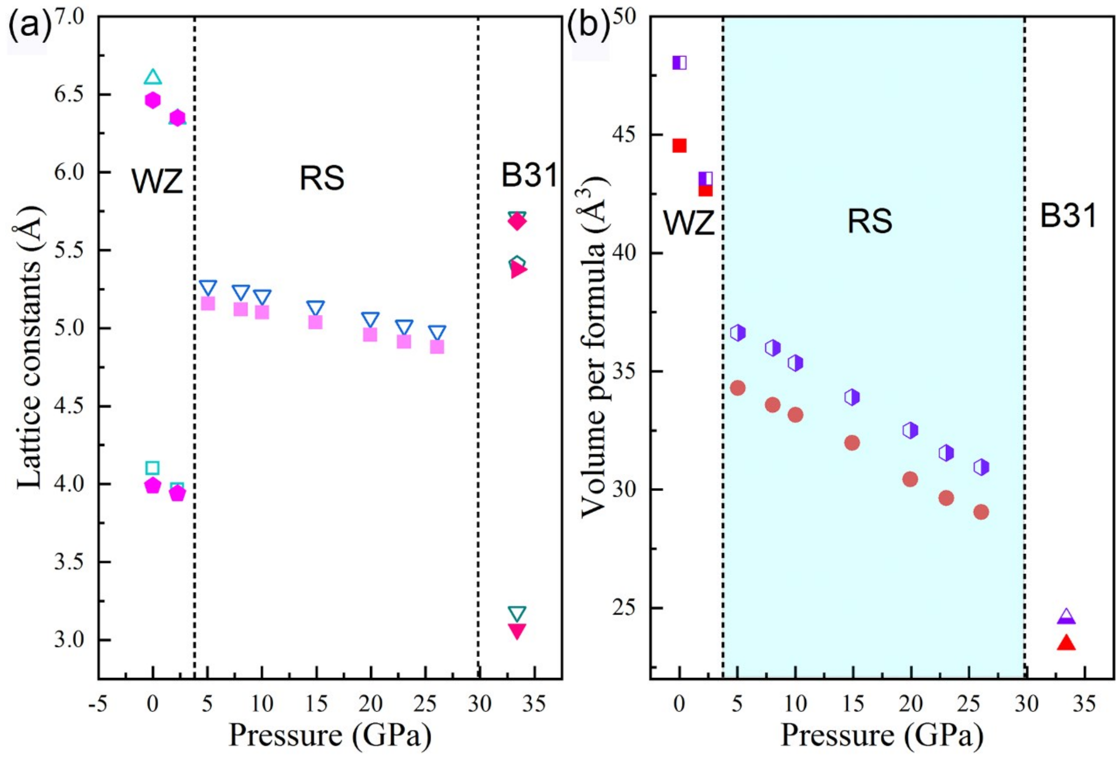

Fig. S3 The lattice constants and lattice volume of MnSe/MnS nanorods as a function of the pressures. (a) Lattice constants (a, b, and c) of WZ-, RS- and B31-type MnSe and MnS as a function of the increasing pressures up to 33.4 GPa. Hollow and solid symbols represent the lattice constants of MnSe and MnS, respectively. (b) Experimental volumes versus applied pressures ranging from 0 to 33.4 GPa for the WZ, RS and B31phases of core/shell MnSe/MnS nanorods. Half-centered and solid symbols indicate the volumes of MnSe and MnS, respectively.

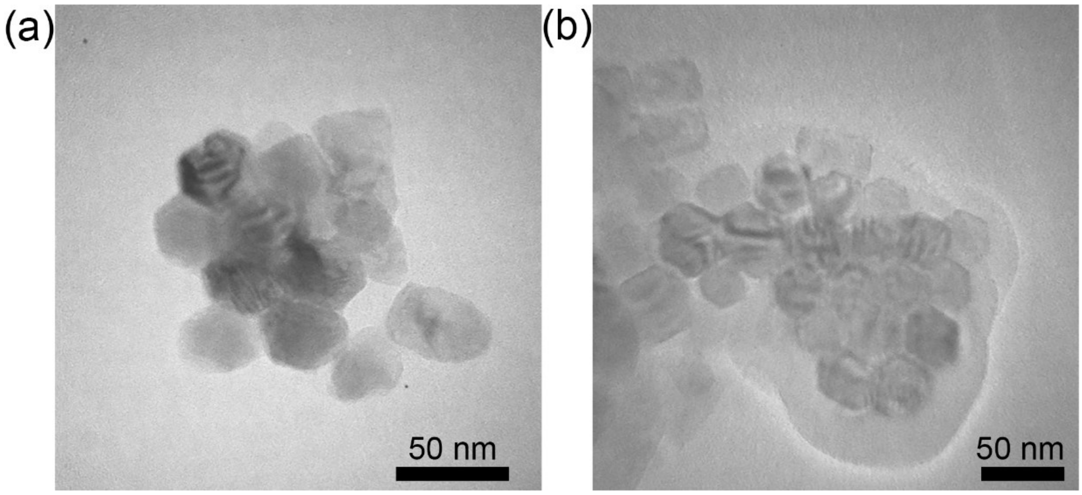

Fig. S4 TEM images of core/shell MnSe/MnS nanorods decompressed from different pressures. (a) TEM image of RS-type core/shell MnSe/MnS nanorods decompressed from 18.0 GPa to 1 atm. (b) TEM image of B31-type core/shell MnSe/MnS nanorods decompressed from 33.4 GPa to 1 atm. TEM images provided evidence for the formation of the new coaxial core shell MnSe/MnS nanorods (RS type and B31 type) with high-pressure phase and preservation of its subsequent dual structure.

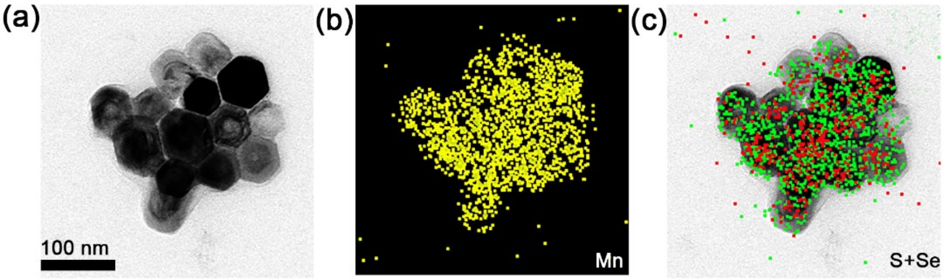

Fig. S5 STEM elemental mapping of B31-type core/shell MnSe/MnS nanorods decompressed from 33.4 GPa to 1 atm. (a) STEM images indicate that the B31-type core/shell MnSe/MnS nanorods still retain the coaxial core shell structure. (b and c) The Energy-dispersive X-ray spectrometry (EDS) mapping further confirms the core−shell nanostructure with Se element in the center and S element in the periphery.

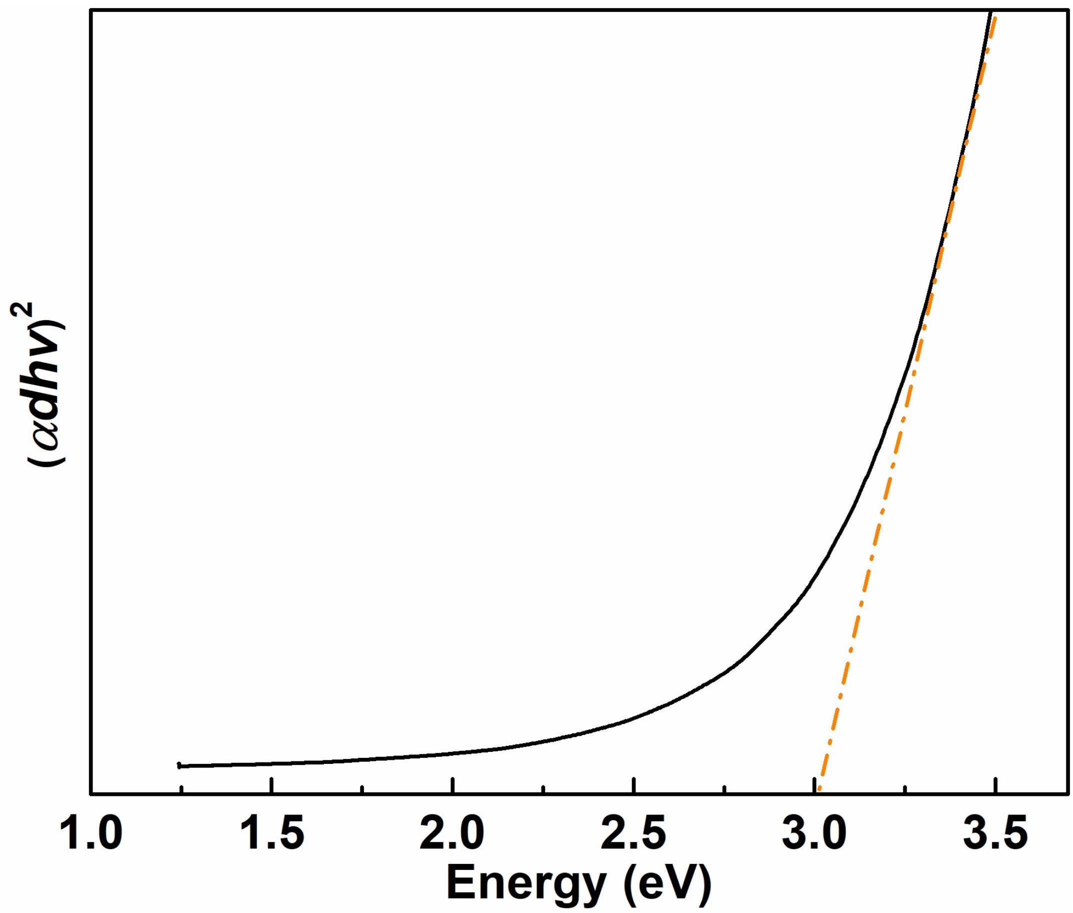

Fig. S6 The band-gap Tauc plots of as-prepared WZ-type core/shell MnSe/MnS nanorods at 1 atm. The band-gap Tauc plots indicate the band gap of as-prepared WZ-type core/shell MnSe/MnS nanorods is 3.03 eV.

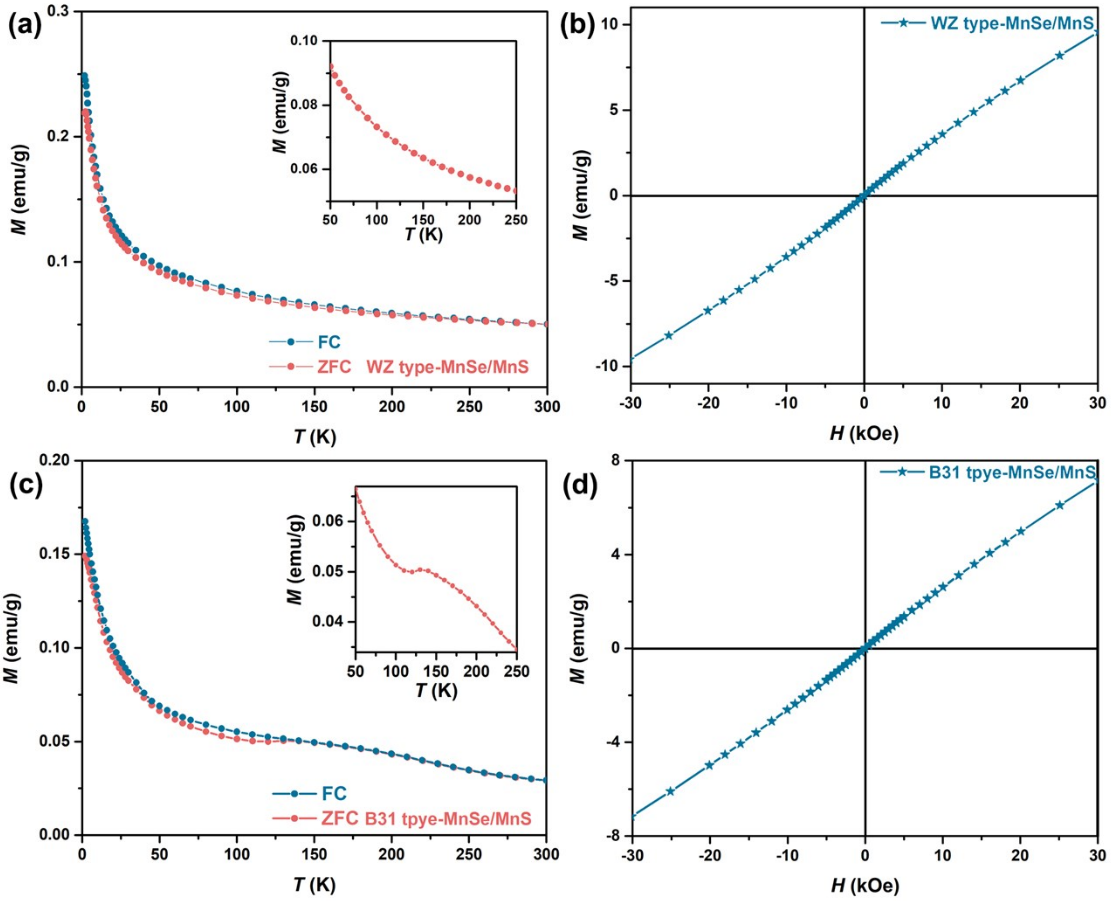

Fig. S7 Magnetic properties of the WZ and B31-type MnSe/MnS nanorods. (a) FC (blue) and ZFC (pink) curves and (b) hysteresis loop of the WZ-type MnSe/MnS nanorods under an applied field of 500 Oe. (c) FC (blue) and ZFC (pink) curves and (d) hysteresis loop of the B31-type MnSe/MnS nanorods under an applied field of 500 Oe. The small peak around 132 K, suggestive of antiferromagnetic ordering, is resolved in the inset of (c) for the B31-type MnSe/MnS nanorods.

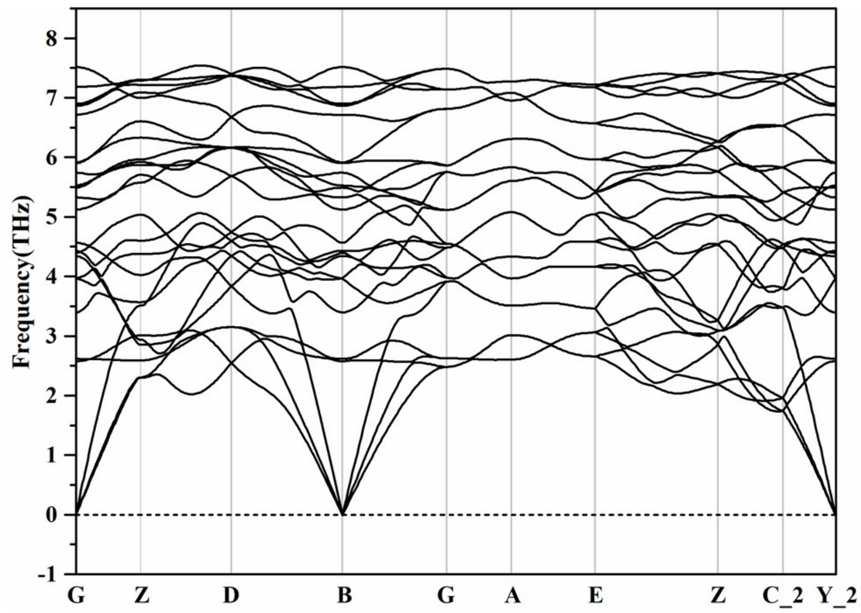

Fig. S8 The phonon spectra of B31-type MnSe. No imaginary phonon frequencies were found in the entire Brillouin zone over the studied pressure range.

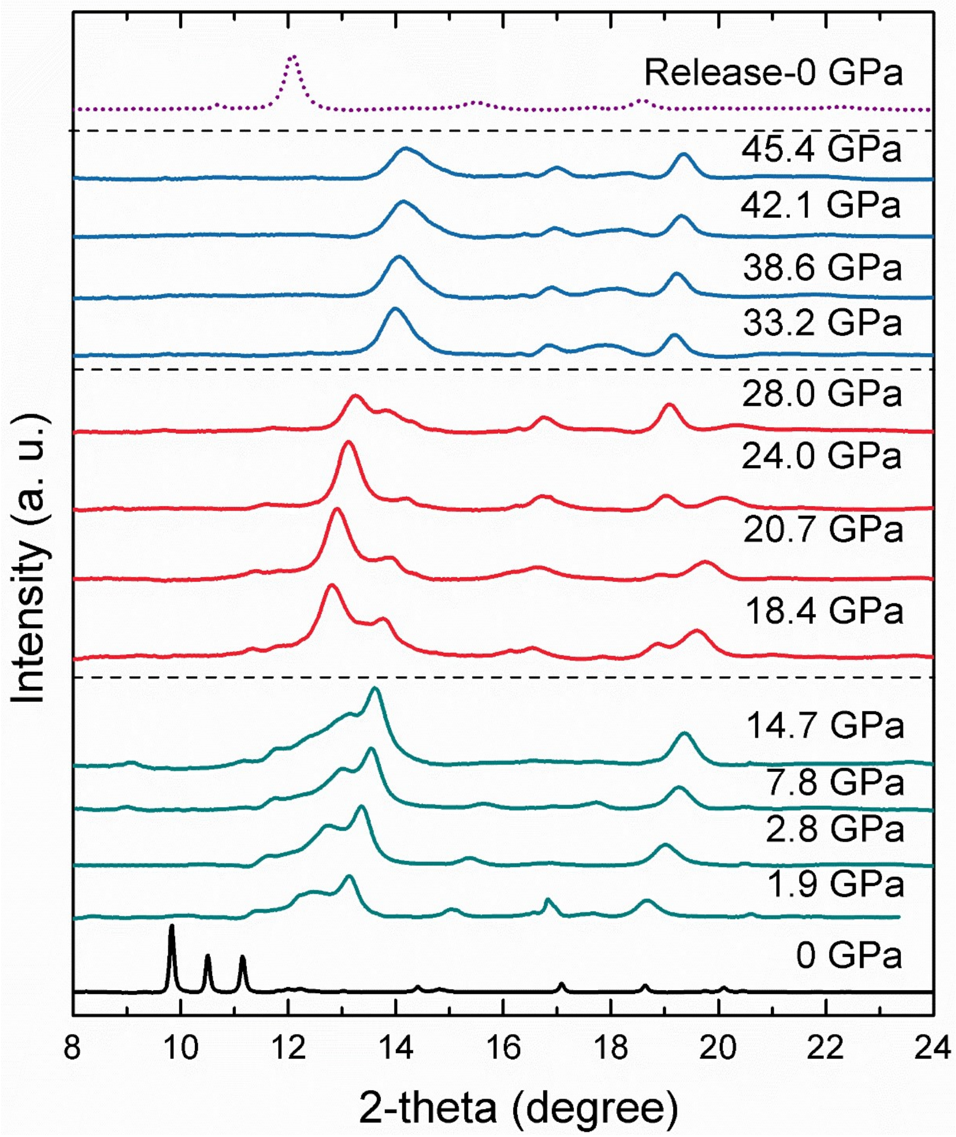

Fig. S9 *In situ* ADXRD patterns of MnSe nanorods during compression and decompression processes. The phase transformation from initial WZ-type MnSe nanorods to RS phase occurred at about 1.9 GPa. The MnSe NCs then changed into B31 phase at above 28.0 GPa, where formation of a HPI phase assigned to a tetragonal distortion structure at applied pressure of 18.4 GPa. The B31 phase MnSe can be obtained when the pressure was released completely.

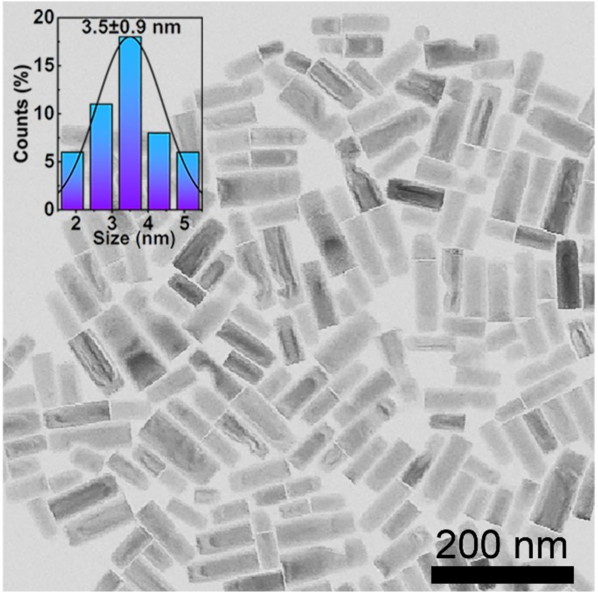

Fig. S10 The shell thickness distribution of WZ-type core/shell MnSe/MnS nanorods with thin shell. The TEM image depicts the high uniformity nature of the as-synthesized core/shell MnSe/MnS nanorods with a shell thickness of 3.5 ± 0.9 nm, based on the analysis of 50 nanorods.

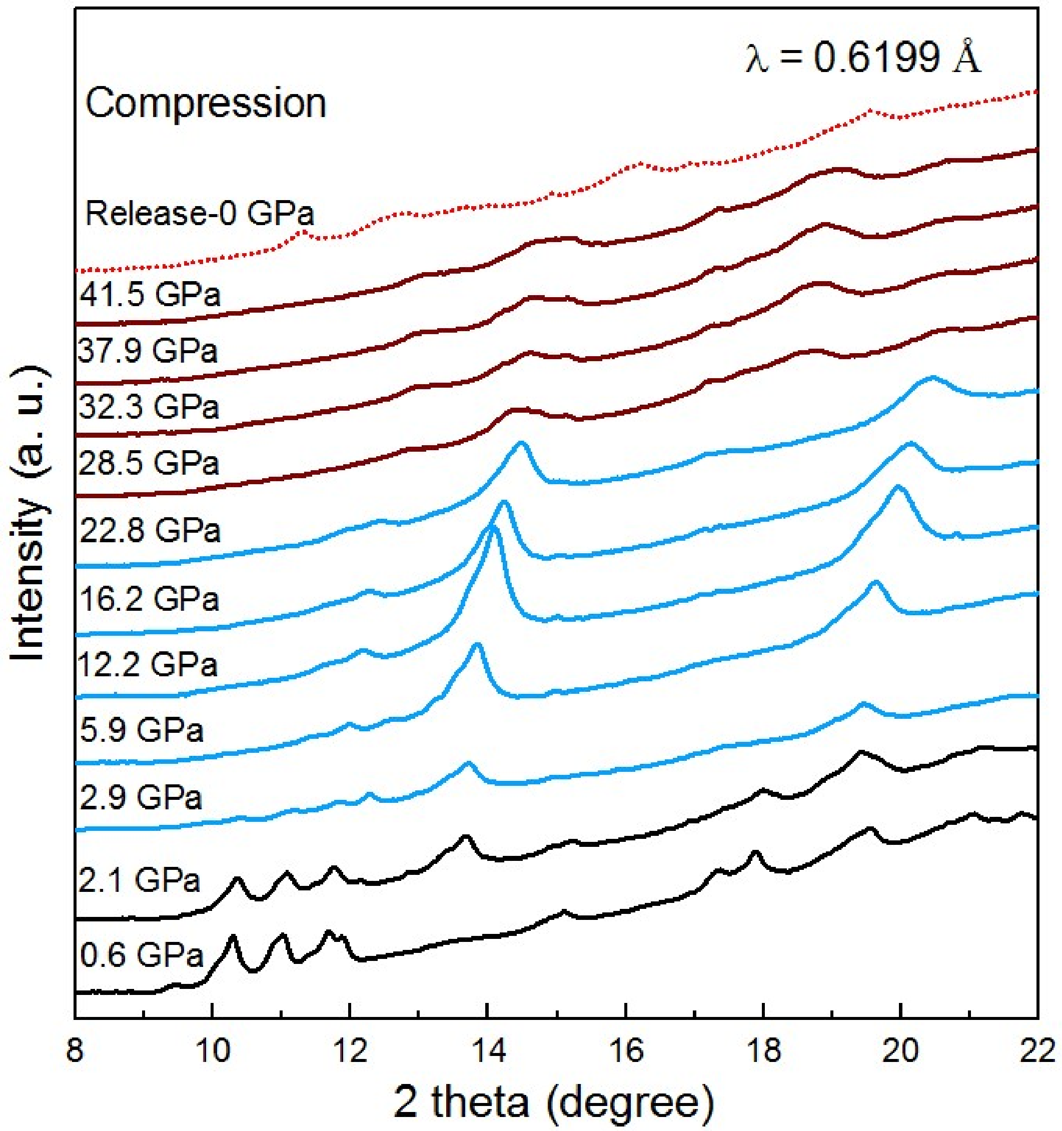

Fig. S11 *In situ* ADXRD patterns of core/shell MnSe/MnS nanorods with thin shell during compression and decompression processes. The phase transformation from initial WZ-type MnSe/MnS nanorods to RS phase occurred at about 2.1 GPa. The MnSe/MnS NCs then changed into B31 phase at about 28.5 GPa. After fully releasing pressure to ambient conditions, pure B31-type core/shell MnSe/MnS can be obtained.

References

[1] B. Zhou, X. Yang, Y. Sui, G. Xiao, Y. Wei and B. Zou, *Nanoscale* 2016, **8**, 8784-8790.
[2] Y. Shang, F. Shen, X. Hou, L. Chen, K. Hu, X. Li, R. Liu, Q. Tao, P. Zhu, Z. Liu, M. Yao, Q. Zhou, T. Cui and B. Liu, *Chin. Phys. Lett.* 2020, **37**, 080701.
